# On the Role of Neural Oscillations Across Timescales in Speech and Music Processing

**DOI:** 10.3389/fncom.2022.872093

**Published:** 2022-06-23

**Authors:** G. Nike Gnanateja, Dhatri S. Devaraju, Matthias Heyne, Yina M. Quique, Kevin R. Sitek, Monique C. Tardif, Rachel Tessmer, Heather R. Dial

**Affiliations:** ^1^Department of Communication Science and Disorders, University of Pittsburgh, Pittsburgh, PA, United States; ^2^Center for Education in Health Sciences, Northwestern University, Chicago, IL, United States; ^3^Department of Speech, Language, and Hearing Sciences, The University of Texas at Austin, Austin, TX, United States; ^4^Department of Communication Sciences and Disorders, University of Houston, Houston, TX, United States

**Keywords:** neural oscillations, cortical tracking, speech tracking, cortical entrainment, neurogenic communication disorders, electrophysiology, speech processing, music processing

## Abstract

This mini review is aimed at a clinician-scientist seeking to understand the role of oscillations in neural processing and their functional relevance in speech and music perception. We present an overview of neural oscillations, methods used to study them, and their functional relevance with respect to music processing, aging, hearing loss, and disorders affecting speech and language. We first review the oscillatory frequency bands and their associations with speech and music processing. Next we describe commonly used metrics for quantifying neural oscillations, briefly touching upon the still-debated mechanisms underpinning oscillatory alignment. Following this, we highlight key findings from research on neural oscillations in speech and music perception, as well as contributions of this work to our understanding of disordered perception in clinical populations. Finally, we conclude with a look toward the future of oscillatory research in speech and music perception, including promising methods and potential avenues for future work. We note that the intention of this mini review is not to systematically review all literature on cortical tracking of speech and music. Rather, we seek to provide the clinician-scientist with foundational information that can be used to evaluate and design research studies targeting the functional role of oscillations in speech and music processing in typical and clinical populations.

## Introduction

The past decade has seen a surge in research on the role of neural oscillations in sensory processing. Neural oscillations are self-sustained rhythmic activity of neural populations that occur at multiple time scales, are generally observed in local field potentials, and may modulate the spiking activity of single neurons (Howard and Poeppel, 2010; Giraud and Poeppel, 2012). They can be seen at rest, and changes in their power and phase can be elicited by external (e.g., sensory stimulation) and internal factors (e.g., self-initiated movements, mind-wandering). Recent research demonstrated that neural oscillations can reliably track ongoing changes in a stimulus (for review, see Haegens and Zion Golumbic, 2018; Meyer, 2018; Myers et al., 2019; Obleser and Kayser, 2019). Consequently, it has been purported that they play an important role in speech and music perception. In this mini-review, we address three main points: (1) oscillatory frequency bands and their functional role, (2) analysis metrics used to study neural oscillations, and (3) the functional relevance of neural oscillations in aging, hearing loss, disorders affecting speech and language (Palana et al., 2022), and music processing. Lastly, we discuss promising methods and future directions for studying neural oscillations.

### Oscillatory Frequency Bands and Their Purported Functional Roles in Speech and Music Processing

Neural oscillations can be observed using a variety of electrophysiological methods with millisecond temporal precision [electroencephalography (EEG), electrocorticography, and magnetoencephalography (MEG)]. Most auditory research on neural oscillations takes advantage of the non-invasive nature of M/EEG to investigate how well neural oscillations align in phase or power with acoustic and linguistic rhythms in speech. This is often referred to as *tracking* or *entrainment*, although the use of these terms is debated (e.g., Obleser and Kayser, 2019; Meyer et al., 2020a,b).

It is unclear whether oscillatory alignment is a result of (1) the summation of delayed, passive, transient, evoked responses to rhythmic stimulus events; (2) active, intrinsic brain oscillations aligning to rhythmic stimulus events; or (3) both (Haegens and Zion Golumbic, 2018; Rimmele et al., 2018; Coffey et al., 2021; cf. Doelling et al., 2019; Zou et al., 2021). In the auditory domain, the mechanistic role of neural oscillations is the subject of ongoing investigation, especially regarding whether oscillations are an emergent property of the auditory system (evoked) vs. an inherent part of that system (intrinsic). A summary of evidence for evoked vs. intrinsic accounts of oscillatory alignment is beyond the scope of this mini-review; we refer the reader to numerous papers on this topic (Haegens and Zion Golumbic, 2018; Lakatos et al., 2019; Poeppel and Assaneo, 2020; cf., Doelling and Assaneo, 2021).

Neural oscillations are typically grouped into frequency bands. These bands arguably play a role in encoding acoustic and linguistic information that unfolds across timescales equivalent to the frequency of the oscillations (Ding et al., 2016; Meyer, 2018; Myers et al., 2019). The slower bands are more engaged in processing information that unfolds across longer periods of time, whereas the faster bands are more engaged for rapidly unfolding information. The delta band (0.5–4 Hz) is thought to encode words, syntactic structures, and prosodic cues in speech and music (Ghitza, 2017; Meyer et al., 2017, 2020a; Keitel et al., 2018; Teoh et al., 2019; Rimmele et al., 2021). The theta band (4–8 Hz) oscillates at a similar rate as syllable production and has been implicated in syllabic processing (Ghitza, 2013; Poeppel and Assaneo, 2020). The alpha (8–12 Hz) and beta (12–25 Hz) bands have been implicated in attention (Wöstmann et al., 2017) and auditory-motor coupling (Fujioka et al., 2012), respectively. The gamma band (25–140 Hz) is hypothesized to encode rapid fluctuations in the auditory signal and be critical for encoding phonetic features (Masuda and Doiron, 2007; Giraud and Poeppel, 2012). Whereas gamma is posited to reflect more bottom-up, lower-level processing of acoustic and phonetic structures in speech, delta and theta may reflect the synthesis of higher auditory and linguistic objects and may modulate gamma activity (Hyafil et al., 2015b). Researchers have proposed a theta-gamma coupling mechanism, with theta oscillations tracking the syllabic structure of speech and providing a temporal frame to group phonetic features encoded by gamma oscillations (Hyafil et al., 2015a; Lizarazu et al., 2019; Hovsepyan et al., 2020).

### Analysis Metrics for Investigating Neural Oscillations

**FIGURE 1:**
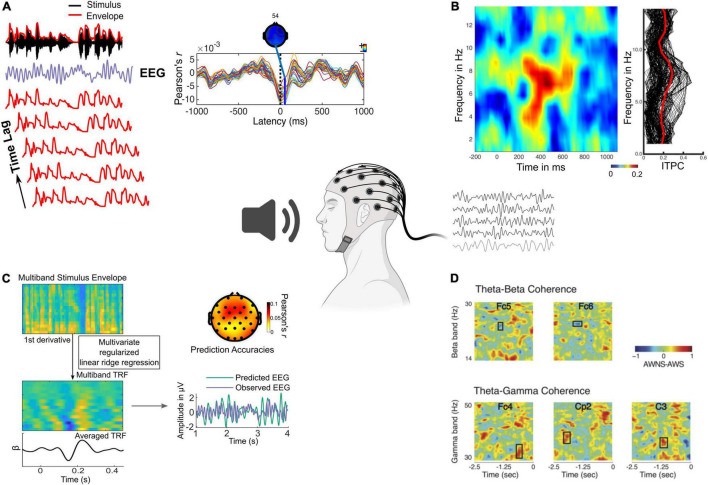
Different analysis metrics used to investigate oscillatory contributions to speech and music perception. **(A)** Cross correlation between stimulus envelope (lag time series) and EEG extracted for sentences. **(B)** Left panel shows time-frequency representation of inter-trial phase coherence for a 6 Hz modulated tone showing stronger values corresponding to the modulation frequency. Right panel shows single trial phase values collapsed across time. **(C)** Multivariate TRF modeling of multiband stimulus envelope to map a kernel function onto the EEG. The observed EEG, multivariate TRF predicted EEG (at a single electrode) and their correlation at each electrode is shown (adapted from Dial et al., 2021). **(D)** Time-frequency representation of cross frequency coupling showing phase amplitude coherence between the theta phase and beta power (above), and theta phase and gamma power (below) in adults who do (AWS) and do not stutter (AWNS) (Sengupta et al., 2019).

A variety of metrics have been used to infer the role of neural oscillations in speech and music processing, including (but not limited to) cross-correlation (Ahissar et al., 2001), multivariate temporal response functions (Crosse et al., 2016), mutual information (Nelken and Chechik, 2007), inter-trial phase coherence (Rimmele et al., 2015), cerebro-acoustic coherence (Peelle et al., 2013), and cross-frequency coupling (Hovsepyan et al., 2020; Figure 1 and Table 1). These metrics convey how well rhythms are tracked by neural oscillations, providing different yet complementary insights into underlying neural mechanisms across frequency bands, acoustic and linguistic rhythms, and brain regions of interest (e.g., cortical vs. subcortical, sensory vs. motor).

**TABLE 1:** Overview of analysis techniques used to study the role of neural oscillations in speech and music processing, the possible inferences that can be drawn when utilizing each technique, and the references in the present mini-review that applied this technique in older adults, clinical populations or music processing (see in-text references for additional context regarding each study).

Technique	Description	Inference drawn from this technique	Studies cited in this mini-review applying this technique	Key findings
Cross-correlation (Figure 1A)	- Correlation between time series of neural oscillations and lagged time series of stimulus features (envelope, periodicity) is assessed to obtain cross-correlation function	- Fidelity of neural response is encoding stimulus features - Latency of neural tracking	Mirkovic et al. (2019), Braiman et al. (2018)	*Mirkovic*: Hearing aid simulator employing a directional microphone led to faster neural processing of speech envelope *Braiman*: Fidelity of cortical tracking of speech envelope in individuals with severe brain injury who successfully performed fMRI mental imagery task is comparable to controls
Multivariate temporal response functions (TRFs) (Figure 1C)	- Regression between time-lagged (to account for neural latency) stimulus features (envelope, phoneme onsets, semantic dissimilarity, etc.) and neural oscillations to predict a temporal response function (TRF) model that explains the mapping between stimulus and neural oscillations - Can be used to reconstruct the stimulus envelope from neural responses	- Time course and source of neural regions tracking stimulus features - Fidelity of representation of stimulus features through model fit - Cannot directly infer entrainment but conveys information about tracking	Di Liberto et al. (2018), Dial et al. (2021), Decruy et al. (2019, 2020), Brodbeck et al. (2018), McHaney et al. (2021), Gillis et al. (2022)	*Di Liberto*: Atypical cortical tracking of phonetic features in children with dyslexia, particularly in the right hemisphere. Magnitude of cortical tracking correlated with phonological processing abilities. *Dial*: Increased cortical tracking of speech envelope at early and late latencies in individuals with logopenic variant primary progressive aphasia vs. age- and hearing-matched controls in theta, but not delta, band Decruy et al. (2019): Supralinear increase in cortical tracking of speech envelope for speech in noise in older adults vs. younger adults. Cortical tracking associated with speech comprehension. Decruy et al. (2020): Larger increase in cortical tracking of speech envelope for attended vs. unattended speech in individuals with hearing loss vs. age-matched controls. *Brodbeck*: Increased cortical tracking of speech envelope in older adults reflects inefficient recruitment of regions outside of primary auditory cortex at early latencies. *McHaney*: Better speech-in-noise comprehension was observed in older adults in whom competing noise showed less deleterious effects on delta band tracking of speech envelope. *Gillis*: Increased cortical tracking of speech envelope and delayed latencies in individuals with hearing loss vs. age-matched controls. Age-matched controls, but not individuals with hearing loss, showed increasingly delayed latencies with greater background noise.
Mutual information	- Assesses statistical dependency between bandpassed stimulus rhythms and neural oscillations - Analysis typically performed at multiple time lags and averaged across time lags - Can also be used to infer statistical dependency between disparate stimulus and oscillatory bands	- Amount of information that is shared between stimulus and neural oscillations in spectral or temporal domains	Zan et al. (2019)	*Zan*: Reduced mutual information between neural responses and stimulus with greater noise in older adults. Older adults show greater reduction in mutual information when competing signals are changed from meaningless to a meaningful speech, suggesting age-related informational loss.
Inter-trial phase coherence (Figure 1B)	- Coherence between the phases of each frequency in every trial estimated while ignoring their absolute magnitude	- Phase locking of neural responses and consistency in phase alignment	Doelling and Poeppel (2015), Sorati and Behne (2019), Yu et al. (2018)	*Doelling and Poeppel*: Musicians showed enhanced cerebro-acoustic coherence across a range of tempi while nonmusicians demonstrated similar coherence only at 1/sec and higher. Degree of coherence correlated with ability to detect pitch distortions. *Sorati and Behne*: Lower inter-trial phase coherence for musicians and non-musicians in delta, theta, and beta bands in audiovisual speech perception. Desynchronization in alpha band for audiovisual speech only in musicians. *Yu*: Inter-trial phase coherence to speech in individuals with autism spectrum disorder increased at earlier latencies and decreased at later latencies vs. controls.
Cerebro-acoustic coherence	- Coherence between stimulus envelope and neural activity obtained using cross-spectral density estimates - Procedure focuses on how individual frequency components of neural oscillations relate to individual frequency components in stimulus envelope - Cannot be used with discrete stimulus features such as word onsets	- Phase-locking of the envelope frequencies and M/EEG spectral components - Informs entrainment in restricted sense but is difficult to separate from evoked activity	Harding et al. (2019), Vanden Bosch der Nederlanden et al. (2020), Mandke et al. (2022), Fiveash et al. (2020), Molinaro et al. (2016)	*Harding*: Cerebro-acoustic coherence to music rhythm increased with years of musical training while response to speech rhythm did not differ as a function of musical training. *Vanden Bosch der Nederlanden*: Under easy listening conditions, neural phase-locking is comparable for spoken sentences vs. sung sentences, but under challenging conditions, better neural phase-locking observed for sung speech, particularly in the theta range *Mandke*: Decreased neural coherence to speech envelope in children with dyslexia in 0-5 Hz and 12-40 Hz range. *Fiveash:* Adults with and without developmental dyslexia showed enhanced stimulus-brain coherence for regular vs. irregular rhythms in music, but individuals with dyslexia did not extract subtle temporal regularities from irregular stimuli. Suggests top-down contributions to neural processing of music. *Molinaro:* Individuals with dyslexia showed impaired entrainment to speech and reduced stimulus-brain synchronization in delta band in primary auditory regions relative to controls.
Cross-frequency coupling (Figure 1D)	- Degree of phase-to-phase or phase-to-power alignment between two different oscillatory frequency bands - Estimated by obtaining the instantaneous phase of a low frequency oscillation and assessing its phase coherence with the instantaneous amplitude envelope of a higher frequency oscillations	- Interaction between oscillations in different bands - Relationships across perceptual timescales or causal relationships between top-down and bottom-up processing	Power et al. (2016)	*Power*: Children with dyslexia showed significantly poorer speech encoding in 0–2 Hz band compared to both chronological and reading age-matched controls. No group differences were found between delta phase and beta power coupling suggesting no differences in sensory-motor coupling between individuals with dyslexia and controls.

### Oscillations in Speech and Music Processing

Neural oscillations can be observed in response to both speech and music. Recent advances in computational modeling have enabled new insights into the mechanisms underlying auditory neural processing. Doelling et al. (2019) modeled MEG responses to music of varying rates, providing evidence for a combination of evoked and intrinsic mechanisms supporting neural processing of musical stimuli. Zou et al. (2021) subsequently utilized this approach with EEG responses to Mandarin narratives. They observed a linear change in phase lag between cortical activity and the speech envelope across frequency bands, suggesting that these oscillations could be modeled as evoked responses. Neural responses to speech and music may thus reflect both evoked and intrinsic oscillatory alignment, depending on the timescale of the stimulus and the oscillatory frequencies of interest. This is of particular interest in music, where the incoming stimulus is generally more isochronous than in speech (Peelle and Davis, 2012; Nolan and Jeon, 2014; cf. Jadoul et al., 2016). However, there is also considerable variability across styles of music and languages [e.g., *syllable-timed* languages (Spanish) feature a more isochronous rhythm than *stress-timed* languages (English, Mandarin); Pike, 1945; cf. Grabe and Low, 2002].

These computational findings add to a growing body of research supporting a role for oscillatory alignment in music processing. Doelling and Poeppel (2015) showed that musicians may have enhanced oscillatory alignment in response to musical rhythms, indicating higher perceptual acuity to subtle spectrotemporal variations. Fujioka et al. (2012) investigated whether MEG beta band oscillations (∼20 Hz) show power and phase coherence to auditory rhythms (i.e., musical beats) across auditory and motor neural systems. Their results suggest that oscillations reflect functional coordination across these systems, referred to as auditory-motor coupling. Fujioka et al. (2015) replicated and extended prior findings specifying that beat encoding by beta band oscillations was influenced by metrical structure (i.e., 4/4 or 12/8 time). Thus, auditory-motor coupling driven by beta oscillations provides an explanation as to why many find it difficult to resist tapping along to one’s favorite song. Further, tests of auditory-motor synchronization to speech rhythm differentiates participants into low and high “synchronizers” (Lizcano-Cortés et al., 2022). However, the precise basis of these distinct behavioral phenotypes have yet to be explored and are a promising avenue for future research in neurotypical and clinical populations.

Harding et al. (2019) studied oscillatory tracking of speech and music using matched stimulus rhythms. They found that individuals with extensive music training showed increased cortical tracking for music vs. speech, whereas tracking in individuals with limited musical training did not differ between music and speech. While such findings point to differences in neural mechanisms supporting speech and music processing, factors like task demands (Devaraju et al., 2021), individual differences (e.g., experience-dependent plasticity; Harding et al., 2019; Sorati and Behne, 2019), and stimulus properties (e.g., “sharpness” of stimulus onset events; Doelling et al., 2019) are important considerations when interpreting study findings.

### Oscillations Across Different Populations

Although most work on cortical tracking of speech has been conducted with neurotypical younger adults, it holds promise as an ecologically-valid tool for assessing speech and language processing in different populations (see systematic review by Palana et al., 2022). For example, Braiman et al. (2018) examined cortical tracking of the speech envelope in individuals with severe brain injury who could not produce overt responses. Individuals who showed evidence of minimally conscious state during a fMRI mental imagery task also showed preserved speech envelope tracking. The benefit of the cortical tracking approach is that it provides an inexpensive, temporally precise measure of rhythmic encoding. Examining cortical tracking of speech is thus an attractive approach for the study of speech perception in neurotypical and clinical populations across the lifespan (Ríos-López et al., 2020; Kolozsvári et al., 2021; Ortiz Barajas et al., 2021).

Recently, increased speech envelope tracking has been observed in the delta-theta range in neurotypical older adults relative to younger adults (Presacco et al., 2016; Brodbeck et al., 2018; Decruy et al., 2019; Broderick et al., 2021) and in individuals with vs. without hearing loss (e.g., Mirkovic et al., 2019; Decruy et al., 2020; Gillis et al., 2022). Similarly, increased speech tracking was observed in the theta range (i.e., syllabic rate) in individuals with logopenic variant primary progressive aphasia (lvPPA), a disorder characterized by impaired phonological processing due to neurodegenerative disease (Dial et al., 2021). This finding was highly reliable across narratives differing in acoustic and linguistic features, further supporting the utility of this method in clinical populations.

In contrast, decreased cortical tracking has been observed in the delta-theta range in children and adults with developmental disorders (e.g., children with dyslexia: Molinaro et al., 2016; Power et al., 2016; Di Liberto et al., 2018; Mandke et al., 2022; adults with dyslexia: Molinaro et al., 2016; Fiveash et al., 2020; children with autism spectrum disorder: Wang et al., 2021; c.f., Yu et al., 2018). Individuals with developmental dyslexia exhibit impaired perception of syllabic stress, prosody, and metrical structure, pointing toward a deviant oscillatory network (Goswami, 2019). This was interpreted in the context of the temporal sampling hypothesis (Goswami, 2011, 2019), which states that delta and theta oscillations in auditory cortex are important for prosody perception and temporal integration at the syllable rate, respectively. The temporal sampling hypothesis is also applicable to other communication disorders like stuttering, wherein individuals exhibit impaired rhythm processing (Wieland et al., 2015), poor temporal resolution (Devaraju et al., 2020), and aberrant neural phase coherence when planning speech utterances (Sengupta et al., 2019).

More recent theories also address atypical rhythm processing in individuals with developmental speech and language disorders. Two such theories are the processing rhythm in speech and music (PRISM) framework (Fiveash et al., 2021) and the atypical rhythm risk hypothesis (ARRH; Ladányi et al., 2020). PRISM highlights the importance of evoked oscillatory alignment to external rhythmic stimuli along with precise auditory timing and sensorimotor coupling. Similarly, ARRH stresses early identification of risk factors (e.g., genetic predisposition) and addressing atypical rhythm processing early. A potentially promising approach for addressing atypical rhythm processing is the use of more song-like speech stimuli, as research has shown that, under challenging listening conditions, neural phase-locking is stronger when speech is sung vs. when it is spoken (Vanden Bosch der Nederlanden et al., 2020). With early identification, individuals may have access to better treatment approaches, leading to better long-term outcomes.

### Does the Magnitude of Cortical Tracking Reflect the Quality of Processing?

As indicated above, both increased and decreased tracking have been observed in older adults and individuals with communication disorders. Some researchers have characterized the relation between tracking and behavior as non-linear, with increased tracking associated with better performance to a certain level, beyond which increases relate to poorer performance (e.g., Schmidt et al., 2021). Increased tracking in older adults and individuals with hearing loss has been interpreted as reflecting the recruitment of regions outside of primary auditory cortex and an imbalance between excitatory and inhibitory mechanisms, leading to over-excitability, and consequently, inefficient processing of acoustic cues in the speech envelope (e.g., Decruy et al., 2019). For example, Brodbeck et al. (2018) found that the largest difference between older and younger adults occurred at a relatively early latency in regions outside primary auditory cortex, suggesting that older adults recruit a larger network of brain regions to process acoustic cues, even at early stages of processing. Increased cortical tracking could also represent a compensatory mechanism to improve speech perception. In fact, increased envelope tracking in older adults (Decruy et al., 2019) and individuals with hearing loss (Decruy et al., 2020) has been related to better speech understanding.

The relation between cortical tracking and speech processing might also be confounded by differential effects across delta and theta bands. In younger adults, Etard and Reichenbach (2019) found increased delta band tracking related to better comprehension. Similarly, McHaney et al. (2021) found that better speech-in-noise comprehension in older adults was related to larger increases in delta band tracking for speech in noise relative to quiet. Dial et al. (2021) found that individuals with lvPPA had increased theta band envelope tracking relative to neurotypical older adults, despite demonstrating worse speech understanding. Thus, increased cortical tracking in the delta band may reflect better comprehension, whereas increased tracking in the theta band may reflect poorer comprehension. However, contradictory evidence exists. Etard and Reichenbach (2019) found that increased tracking in the theta band in younger adults positively related to perceived speech clarity. Drawing strong conclusions about the unique roles of the delta and theta bands in speech processing is difficult (partially because many studies examine the delta-theta *range* or an even broader range; e.g., Gillis et al., 2022). Future work should examine these bands separately and, perhaps, instantiate non-linear analysis methods to further elucidate the role of cortical tracking in speech and music processing.

## Discussion

Neural oscillations play a critical role in speech and music processing, contributing to our understanding of these processes in various populations. In this mini-review, we presented an overview of neural oscillations, methods for studying them, and their functional relevance to aging, hearing loss, speech and language disorders, and music processing. In the following, we discuss methodological advances that may further elucidate the role of oscillations in auditory processing.

To date, neural oscillations have been studied using M/EEG with high temporal but poor spatial precision. Recent advances in neuroimaging methods with good spatial precision enable acquisition with higher temporal resolution (≤1 s) (Lin et al., 2013; Lewis et al., 2016). For instance, a recent fMRI study with 1 Hz sampling found that hemodynamic responses tracked the envelope of attended speech, particularly in right hemisphere non-primary auditory cortex (Hausfeld and Formisano, 2021). A related technique, functional near-infrared spectroscopy (fNIRS), has superior temporal resolution, higher motion tolerance, and fewer contraindications (e.g., cochlear implants) than fMRI. This makes it a strong candidate for research seeking to localize brain areas where typical/atypical oscillatory mechanisms exist in various populations.

Neuroimaging methods can be applied in tandem, compensating for individual methods’ shortcomings. For example, pairing EEG and fMRI allows for temporally precise localization of neural patterns (Philiastides et al., 2021; cf. Chang and Chen, 2021; Scrivener, 2021). Combined EEG-fMRI has already been applied in research on hemispheric specialization of neural oscillations in dyslexia (Schulz et al., 2008; Lehongre et al., 2013) and in neurotypical individuals to examine cortical tracking of speech in noise (Puschmann et al., 2017). To our knowledge, combined EEG-fMRI has yet to be applied to cortical tracking in clinical populations. This could improve our understanding of the loci of neural lesions contributing to functional differences in cortical tracking, further informing treatment approaches (Lehongre et al., 2013).

Another promising technique is transcranial alternating current stimulation (tACS), which uses a signal matched to different rhythms in incoming stimuli to stimulate brain areas involved in perception. tACS provides a direct method for establishing a causal relationship between external rhythms and neural oscillations. Several studies demonstrated improved acoustic and speech processing following tACS (Jones et al., 2020; Keshavarzi and Reichenbach, 2020; Erkens et al., 2021; Keshavarzi et al., 2021; for recent reviews, see Riecke and Zoefel, 2018; Nooristani et al., 2021). Researchers have also argued for a relation between aberrant cortical tracking of speech and speech-in-noise difficulties in individuals with hearing loss (Fuglsang et al., 2020; Vander Ghinst et al., 2021). tACS is thus a promising method for studying neural oscillations and may improve perception in clinical populations. Moreover, combined M/EEG-tACS may further elucidate intrinsic vs. evoked accounts of neural oscillations (e.g., van Bree et al., 2021).

Beyond multimodal neuroimaging, advances in computational approaches (Doelling et al., 2019; Accou et al., 2021; Guest and Martin, 2021) provide exciting avenues for research on cortical tracking of speech and music and a deeper investigation into the unique contributions of different oscillatory bands. For example, researchers recently utilized neural networks to model predictions based on theta-gamma coupling in syllable recognition and speech prediction (Donhauser and Baillet, 2020; Hovsepyan et al., 2020). Additionally, improvements in natural language processing have resulted in stimulus models representing higher-level linguistic processing, allowing researchers to examine cortical tracking of features like semantic dissimilarity (Broderick et al., 2018).

Research in clinical populations and across the lifespan has just begun to explore cortical tracking of linguistic features at sublexical (e.g., phonetic features), lexical (e.g., word entropy), semantic (e.g., semantic dissimilarity), and syntactic (e.g., surprisal based on part of speech) levels (e.g., Mesik et al., 2021). Such investigations could elucidate the mechanistic underpinnings of impaired processing and assist in identifying deficits in clinical populations, avoiding confounds associated with traditional neuropsychological assessment (e.g., overt responses). This, in turn, could provide treatment targets and a way to assess treatment-induced changes. In sum, the study of neural oscillations provides a unique window into the brain through which we can assay the neurobiological computations supporting speech and music processing in neurotypical and clinical populations. The rapid evolution of this field is promising for basic and applied research and has immense potential for steering neurobiologically informed treatment methods.

## Author Contributions

All authors listed have made a substantial, direct, and intellectual contribution to the work, and approved it for publication.

## Conflict of Interest

The authors declare that the research was conducted in the absence of any commercial or financial relationships that could be construed as a potential conflict of interest.

## Publisher’s Note

All claims expressed in this article are solely those of the authors and do not necessarily represent those of their affiliated organizations, or those of the publisher, the editors and the reviewers. Any product that may be evaluated in this article, or claim that may be made by its manufacturer, is not guaranteed or endorsed by the publisher.
